# Perception-based analysis of climate change effect on forest-based livelihood: The case of Vhembe District in South Africa

**DOI:** 10.4102/jamba.v8i1.271

**Published:** 2016-07-29

**Authors:** Chidiebere Ofoegbu, Paxie W. Chirwa, Joseph Francis, Folarannmi D. Babalola

**Affiliations:** 1 Forest Science Postgraduate Programme, University of Pretoria, South Africa; 2 Institutes for Rural Development, University of Venda, South Africa; 3 Department of Forest Resources Management, University of Ilorin, Nigeria

## Abstract

Forests are vulnerable to climate change and are also major sources of livelihood for many rural households in Africa. This study examines rural people’s perceptions of climate change impacts on forest-based livelihoods using rural communities of Vhembe District in South Africa as a case study. The study was based on the principles of perceived impact-based assessment, and sustainable livelihoods framework. Using the stratified proportionate random sampling procedure in combination with weighted Enumeration Area for the selected communities, 366 households were chosen and interviewed. Data analysis involved computing frequencies and conducting the Chi-square, binomial tests and binary logistic regression analysis. The respondents identified erratic rainfall, extreme temperature, extreme drought and flooding as key climatic events in their community. But not all identified key climatic events were perceived to constitute risk to forest products and forest-based livelihood. Only extreme drought was indicated to constitute risk to availability of forest products. In addition, the binary logistic regression showed a significant difference (*p* < 0.05) in the perceived risk of climate change to the availability of essential forest products across the three municipalities. Hence the need for forest development initiatives that target vulnerable forest products per community as a means of enhancing resilience of forest-based livelihood to climate change impacts in rural community development in South Africa.

## Introduction

Households’ livelihoods in most rural communities in South Africa, like elsewhere in Africa, are highly dependent on forest resources (Charnley 2005; Clarke & Isaacs 2005; Davies, Oswald & Mitchell 2008; Malone & Rovere 2012; Hachileka 2009; Mertz *et al*. 2009), with farming, animal husbandry, and harvesting and trade in forest resources being the dominant livelihood activities (Chamberlain *et al*. 2005; Kyei 2011; Vhembe 2013). Forest resources are in essence highly crucial for rural development in South Africa. However, observed and predicted impact of climate change is projected to have an extensive range of consequences, many of which represent major threats, for example droughts, flash floods and decline in crop productivity, among others (Bryan *et al*. 2009; Capstick 2012; Chinara *et al*. 2013; Kalinda 2011; Mengistus 2011; Mertz *et al*. 2009; Sarah *et al*. 2012). This impact also poses significant threat to forests, livelihoods and rural development which might result into increased intensity of poverty.

Key sectors that are vital for rural development in South Africa such as forests, water resources, tourism and agriculture are vulnerable to climate change impacts (IPCC 2007; Lazo, Kinnell & Fisher 2000). Specifically, there is growing evidence that climate change impacts will diminish the capacity of forests to provide goods and services with serious implication to the livelihood of forest-based rural communities (Okali 2011). Moreover, close proximity and strong linkages to climate-sensitive environs of forest-based communities make them uniquely vulnerable to climate change (Boon & Ahenkan 2012; Davidson, Williamson & Parkins 2004). Because of their geographical location, forest-based communities are extremely exposed to recurrent environmental threats like forest fire, pest and disease out-break, and strong wind (Davidson *et al*. 2004); these risks can be exacerbated by climate change. In addition to being exposed to climate change impacts, forest-dependent rural communities in South Africa are also faced with socioeconomic challenges such as high unemployment rate, poverty and low economic development opportunity (Vhembe 2013).

Several authors have investigated the impact of climate change on the forestry sector. Williamson Parkins and McFarlane (2005) examined forestry experts’ perceptions of climate change impacts on forest ecosystem and forest-based communities. Davidson *et al*. (2004) reported that forest fire activity is on the increase in Canada. Chinara *et al*. (2013) observed that possible changes in climate can make current livelihoods unsustainable, resulting in deeper poverty and a shift into poverty of those who are currently not poor. In the same vein, Kalinda (2011) reported that climate change is a major challenge towards attainment of sustainable development in most African countries and can adversely affect poverty reduction efforts in the rural communities. Furthermore, Mengistus (2011), Sarah *et al*. (2012) and Mertz *et al*. (2009) predicted that climate change will have negative effects on forest resources and rural livelihood, which will pose a significant threat to Africa’s attainment of sustainable development.

Although perception of climate change impact is increasingly gaining recognition in disaster assessment (Williamson *et al*. 2005), concerns about rural people’s ability to perceive climate change impact is equally increasing. Maddison (2007) is of the view that rural farmers may take time to realise that unusual weather represents a permanent shift in the climate. Claas *et al*. (2011) argue that the majority of people rate the impact of climate change on an abstract, cognitive level which might lead to an underestimation of the hazards of climate change. However, efforts towards effective resilience improvement strategies and formulation of climate change intervention policies are difficult without an understanding of individuals’ perception of climate change impacts (Alexa *et al*. 2010). As noted by Davidson *et al*. (2004), people’s perceptions of impacts will influence how and when households might start to take specific actions to adapt to climate change. An understanding of rural people’s perception of climate change impact on their livelihood is therefore crucial to improving the people’s resilience to climate change impact (Hansen *et al*. 2007). This is also essential in assuring that efforts to address climate change impacts correspond to actual concerns (Chinara *et al*. 2013).

Most studies on the impacts of climate change on forests and rural development in South Africa have focused on biophysical impacts vis-à-vis temperature rise and erratic rainfall, among others (Davidson *et al*. 2004; Lindner *et al*. 2008; Naidoo, Davis & Van Garderen 2013; Turpie & Viser 2013), and forest ecosystem functioning (Fairbanks & Scholes 1999), and there economic implications to society, while human dimensions including socioeconomic implications of climate change at rural community level have been overlooked. As reported by Piya, Maharjan and Nira (2012), climate change manifestations in any community are particularly unique to economic, ecological and social characteristic of such community. In fact, there is increasing scientific evidence that highlights the importance of understanding the context-specific impacts of climate change (Claas *et al*. 2011; IPCC 2007). Hence, the need to understand the susceptibility of forest-based communities to climate change impacts in South Africa. This understanding is fundamental for expounding climate change manifestations to a level where practical local intervention action is possible (Chinara *et al*. 2013).

The perception of climate change impacts at household level has not received much attention of researchers in South Africa. Most importantly, the effects of climate change on local contexts, particularly in rural forest-based communities, are poorly understood (Turpie & Visser 2013; Williamson *et al*. 2005). This knowledge gap is pertinent for the forestry sector which forms the bedrock of most of rural livelihoods in the country (Chigavazira 2012; Nzuma *et al*. 2010; Quinn *et al*. 2011). This study therefore sought to address this gap by examining perceptions of local people on climate change impacts and pathways through which forest-based livelihoods in rural communities are impacted by climate variability and extreme weather events. It evaluated climate change impacts in the context of perceived exposure and sensitivity of essential forest products to climate variability and extreme event. The case study communities were forest-based rural communities in Vhembe District of South Africa.

With respect to the forgoing, the study intended to provide answers to the following questions with respect to climate change impacts in rural communities of South Africa:

- What climatic variability and extreme event have been observed by the people over the past years?
- How do they perceive the effect of this climatic phenomenon (positive and negative) on forest-based livelihoods in their communities?
- Are there any forest products that are incurring more climate-associated threats? Why is this so and what are the implications for sustainable livelihood in the communities?

Findings of this study could initiate formulation of policies and development of initiatives towards effective and sustainable climate change intervention in rural communities of South Africa.

## Methodology

### Description of study area

**FIGURE 1:**
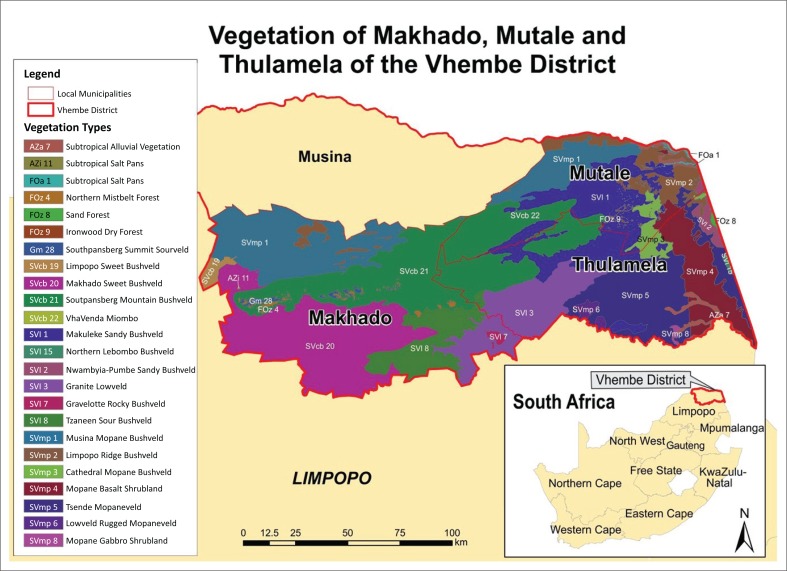
Map of Vhembe District.

The study was conducted in Vhembe District Municipality, Limpopo province of the Republic of South Africa (22° 56 S, 30° 28E), as shown in Figure 1. Vhembe is one of the five districts of Limpopo province of South Africa. The district contains the following local municipalities: Thulamela, Makhado, Mutale and Musina. The district has a total population of 1 199 886 with 54.40% females and 45.60% males. The majority of the population resides in Vhembe (48.72%) and Makhado (41.43%), the remaining population are distributed across Mutale (6.58%) and Musina (3.28%) (Statistics South Africa Census 2011). The main languages spoken are Tshivenda (69.00%) and Xitsonga (27.00%). To cover the various representations of forest-based livelihood types in the district, we use the criteria of vegetation types, and dominant livelihood strategy to select three municipalities: Makhado, Mutale and Thulamela for this study. In each of the selected municipality, we further selected a replicate of seven rural communities. This gave a combined total of 21 rural communities, which were then surveyed for this study. A total of 366 households were selected from the study’s 21 rural communities using stratified proportionate random sampling procedure. For questionnaire administration, respondents in a selected household were supposed to (1) be more than 20 years old and (2) have lived in their community for more than 5 years.

### Analytical framework

The questionnaire used for the assessment of climate change impacts was adopted from the Climate Risk Assessment Guide developed by United Nations Development Programme (UNDP) Central Asia Climate Risk Management Program (UNDP 2013). The Guide uses a modification of the Sustainable Livelihoods Framework to define climate change impacts by looking at how short or long-term climatic events can affect different types of forest products used for livelihoods in the study communities. The use of questionnaires enabled identification of the community’s prioritised climatic hazard and comparisons of the results within as well as between municipalities.

The first part of the questionnaire focused on the identification of climatic and extreme weather events occurring in the community. We used meteorological information and literature to identify climatic events occurring in Vhembe District (Davis *et al*. 2010). Respondents were then asked to tick the events that their household and livelihoods are exposed to. We further explored respondents’ perceived impact of these events to forests and forest-based livelihood in their community. A 4-point Likert scale (1 = No effect, 2 = Resources has become scarce, 3 = Increased difficulty in harvesting resource and 4 = Resource has become expensive) was used to analyse perceived effect and sensitivity of essential forest product to climate change impacts. A 3-point Likert scale was used (1 = No effect, 2 = temporary reduced access 3–4 months and 3 = reduced access for extended period of time of up to 5 months and above) to assess climate change effect on access to forests. A similar rating technique has been used by Asherleaf (2012), Lazo *et al*. (2000) and Williamson *et al*. (2005) in analysing climate change effect on forest-dependent communities in Canada and by Badjeck *et al*. (2010) in analysing the impacts of climate variability and change on fishery-based livelihoods.

### Data analysis

The weighting adjustment technique was applied to correct for possible problems of either over- or under-representation of variables (Bethlehem 2015). The sample was weighed against the actual population to arrive at the weighted sample. Thereafter, the weighted data were imported into the Statistical Package for Social Sciences (SPSS) version 23.0. Data analysis involved computing frequencies and conducting the Chi-square, binomial tests and binary logistic regression analysis.

Binomial test was used to analyse perceived increase and decrease of each climate and extreme events occurring in each municipality in order to identify significant (priority climate hazard identification analysis) key climatic events for each municipality (Berg 2014). The different climate variability and extreme weather events studied were erratic rainfall, extreme temperature, serious flooding, extreme drought, strong wind and incidence of hailstones. In this study, climate variability refers to the natural fluctuations of the climate system while an extreme weather event is a weather event that is significantly different from the average or usual weather pattern. We also used binomial test to analyse the ‘yes’ and ‘no’ responses of climate variability and change effect on forest products (Berg 2014).

We further analysed the sensitivity of essential forest products used for livelihood in the study communities to the identified key climatic events in order to ascertain whether there were any forest products that were perceived to be incurring more climate-associated threat. A binary logistic model was used to estimate sensitivity or relationship between perceived changes in climatic event and perceived effect of climate variability and change on essential forest products used by communities (Maddala 1983). Odds ratios were used to measure the magnitude of strength of association or non-independence between two binary data values. The sensitivity of the essential forest products, woody products (firewood, charcoal, timber or construction wood) and non-woody products (mushrooms, medicinal plants, thatch grass, bush meat or wild edible insects, wild fruits or vegetables) were tested. Perceived change in climatic event was framed as binary-choice models which assumed that respondents’ perception of the increase or decrease in woody and/or non-woody products dictated their perceived increase or decrease in climatic events. The perceptions are dependent on identifiable characteristics.

Let *T_i_* represent a dichotomous variable that equals 1 if respondent perceived increase in climatic event over the years and 0 if the perception was decrease.

The probability of perceiving increase in climatic event, Pr (*T_i_ =* 1), is cumulative density function *F* evaluated at *X_i _β*, where *X_i_* is a vector of explanatory variables and β is a vector of unknown parameter (Maddala 1983). This cumulative density function was modelled using logistic probability function, which has the following form:

$$\text{The probability of perceived increase}=\text{Pr}(T_{i}=1)=\frac{\exp(X_{i}\beta )}{1+\exp(X_{i}\beta )}$$ [Eqn 1]

The estimated model was:

$$\begin{array}{l} \text{Perception of increase in climatic event}={\text{b}}_{0}+{\text{b}}_{1}(\text{GND})+{\text{b}}_{2}\\ (\text{EMP})+{\text{b}}_{3}(\text{FIRE})+{\text{b}}_{4}(\text{CHAR})+{\text{b}}_{5}(\text{TIM})+{\text{b}}_{6}(\text{MUSH})+{\text{b}}_{7}\\ \left(\text{MEDI})+{\text{b}}_{8}(\text{WILD})+{\text{b}}_{9}(\text{FODD})+{\text{b}}_{10}(\text{THAT})+{\text{b}}_{11}(\text{HON}\right) \end{array}$$ [Eqn 2]

The dependent variable is the perception of occurrence of a climatic event, which takes on the value of ‘1’ if the respondent perceived increase in climatic event and ‘0’ if decrease is perceived. The following climatic events were separately analysed: erratic rainfall, extreme temperature, extreme drought and flooding. Explanatory variables and justification are discussed below.

Gender (GND) is a dummy variable (male = 1; female = 0); employment status (EMP) (employed = 1; not employed = 0); firewood (FIRE) measures perceived effect of climatic event on firewood availability in the community (reduction in quantity = 1; no effect = 0).

Charcoal (CHAR) measures perceived effect of climatic event on charcoal availability in the community (reduction in quantity = 1; no effect = 0); timber (TIM) measures perceived effect of climatic event on timber (reduction in quantity = 1; no effect = 0); mushroom (MUSH) measures perceived effect of climatic event on mushroom collection from the forest in the community (reduction in quantity = 1; no effect = 0); medicinal plants measure perceived effect of climatic event on medicinal plants collection from the forest in the community (effect of climatic event = 1; no effect = 0); Wild fruits and vegetables measure perceived effect of climatic event on wild fruits and vegetables collected from the forest in the community (effect of climatic event = 1; no effect = 0). Fodder for livestock measures perceived effect of climatic event on livestock fodder in the community (effect = 1; no effect = 0); thatch grass (THAT) measures perceived effect of climatic event on thatch grass collection from the forest in the community (effect = 1; no effect = 0); and honey (HON) measures perceived effect of climatic event on honey collection from the forest in the community (effect = 1; no effect = 0).

## Results and discussions

### Demographic characteristics of respondents

The results of demographic information of the households presented in Table 1 show that majority of the respondents were within the age range of ≤ 35–69. Thulamela had the smallest proportion of population within the age limit of ≥ 70. Furthermore, women were the majority in all the municipalities (71.6% – 83.3%), largely because men migrate to urban areas for work and send remittances to their families in the rural area. Majority of the respondents in Makhado (64.6%) and Mutale (73.4%), and fewer respondents in Thulamela (45.5%) did not have formal education. Length of residency of most respondent was in the range of 1–5 years (Makhado), 11–15 years (Mutale) and 20 years and above (Thulamela).

**TABLE 1:** Demographic profile of respondents.

Demographic characteristics	Makhado (%)	Mutale (%)	Thulamela (%)
**Age (years)**	**-**	**-**	**-**
≤ 35	17.8	15.5	34
36–47	18.5	12.7	27
48–58	21	18.2	20
59–69	21.7	27.3	14
≥ 70	21	26.4	5
**Gender (%)**	**-**	**-**	**-**
Male	16.7	28.4	20
Female	83.3	71.6	80
**Length of residency**	**-**	**-**	**-**
1–5	23.1	16.4	26
6–10	18.6	18.2	19
11–15	22.4	29.1	12
16–20	19.9	24.5	16
More than 20	16	11.8	27
**Highest level of education (%)**	**-**	**-**	**-**
No formal education	64.6	73.4	45.5
Grade 11 or lower	16.5	17.4	23.2
Grade 12 (Matric, std. 10)	12.0	2.8	21.2
Post-matric diploma	3.8	4.6	4
Baccalaureate degree (s)	1.9	0	1
Postgraduate degree(s)	1.3	1.8	5.1

### Perception of climate change impacts

Results in Table 2 show that the majority of respondents in Makhado (59.2%), Mutale (70.0%) and Thulamela (58.0%) perceived that incidence of erratic rainfall had increased. In addition, the Bonferroni test shows that these perceptions do not differ significantly. The comparison analysis of perceived increase and decrease in erratic rainfall events in the three municipalities showed that there was a significant increasing trend in erratic rainfall event in all municipalities (*p* = 0.00). Similarly, we observed significant increasing trends in the occurrence of incidence of extreme temperature and flooding event in all the municipalities (*p* = 0.00). This is consistent with the findings of Davies *et al*. (2008) where mounting evidence of increase in climate variability and climate change was observed in many of the climatic zones of South Africa. Similarly, Naidoo *et al*. (2013) observed clear evidence of rising temperatures at the local level in Vhembe District and semiarid regions of South Africa.

**TABLE 2:** Perceived increase and decrease in climatic events in the study communities.

Climatic event	Response	Proportion of respondents (%) in		

		Makhado (*n* = 156)	Mutale (*n* = 110)	Thulamela (*n* = 100)
Erratic rainfall	Increasing	59.2^a^	70.0^a^	58.0^a^
	Decreasing	29.9^a^	22.7^b^	36.0^c^
	No change	10.8^a^	7.3^a^	6.0^a^
	Binomial test	0.00*	0.00*	0.00*
Extreme temperature	Increasing	75.8^a^	85.3^a^	74.0^a^
	Decreasing	20.4^a^	13.8^a^	12.0^a^
	No change	3.8^a^	0.9^a^	14.0^b^
	Binomial test	0.00*	0.00*	0.00*
Flooding	Increasing	47.8^a^	64.2^b^	41.4^a^
	Decreasing	40.1^a^	20.2^b^	34.3^a,b^
	No change	12.1^a^	15.6^a,b^	24.2^b^
	Binomial test	0.00*	0.00*	0.00*
Serious drought	Increasing	51.6^a^	72.5^b^	27.3^c^
	Decreasing	36.3^a^	20.2^b^	51.5^c^
	No change	12.1^a,b^	7.3^b^	21.2^a^
	Binomial test	0.00*	0.00*	0.00*

Each superscript letter denotes a subset of community categories whose row proportions do not differ significantly from each other at the 0.05 level. Binomial test analysis of increasing and decreasing responses by respondents.

*, significant at 0.05; ns = not significant at 0.05.

In contrast, we only observed a significant increasing trend in the occurrence of incidence of serious drought in Makhado and Mutale (*p* = 0.0000). In Thulamela, the incidence of serious drought was observed to be significantly decreasing (*p* = 0.00). The observed differences in the trend of occurrence of serious drought event in the three municipalities can largely be attributed to the state of water supply facilities in the municipalities. In Thulamela, tap water supply facility is widespread in all the rural communities, and there is a high level of satisfaction with water facility. Hence the effect of serious drought is substantially managed and less felt by the people. However in Mutale and Makhado where there was a high level of dissatisfaction with water supply facility, the effect of serious drought is greatly felt by the people. The situation is more alarming in Mutale, which is located in the arid region characterised with increased water stress challenges.

Furthermore, binomial test analysis of respondents’ prioritised perception of the risk of climate variability and extreme events in their community showed that all four investigated climatic events (erratic rainfall, extreme temperature, serious drought and flooding) were prioritised as key climatic events for Makhado and Mutale municipalities. However for Thulamela only erratic rainfall, extreme temperature and extreme wind were prioritised as key climatic impact factors. Consequently, we postulate that forest-based livelihoods in Mutale and Makhado may be more at risk to climate variability and change than those in Thulamela. Increased occurrence of these climatic events can have significant impact on forests and forest-based livelihoods in the study communities. As observed by Mpandeli and Maponya (2013) and Lindner *et al*. (2008), rising temperatures and decreasing rainfall will lead to increased occurrence of drought periods in semiarid regions of Vhembe. This occurrence may lead to an increase in fire risks.

### Perceived effect of climate variability and change on access to forest

There are complex ways through which climatic variability and change can either directly or indirectly affect forests and forest-based livelihoods. Table 3 shows results of analysis of perceived effect of climate variability and change on access to forest for essential forest products used for livelihood in the study communities. There were generally significant differences across the municipalities in perceived effect of climate variability and change on access to forest products (*p* = 0.000). For all the four climatic events analysed, in Makhado the response in access to the forest resource was similar (28% – 42%). However, for Mutale there tended to be a temporarily reduced access to the forest resource as a result of all the climatic events (73% – 91%). Interestingly, in Thulamela more people (50% – 58%) felt that there was no effect on access to forests. The difference in perceived effect of climate variability and change on access to forest in the municipalities can largely be attributed to the availability of more forests around and within study communities in Thulamela area.

**TABLE 3:** Perceived effect of climate variability and change on access to forest.

Climatic event	Responses	Proportion of respondents (%) in		

		Makhado (*n* = 156)	Mutale (*n* = 110)	Thulamela (*n* = 100)
Erratic rainfall	No effect	30.1^a^	24.8^a^	58^b^
	Temporary reduced access (3–4 months)	34^a^	73.4^b^	37^a^
	Extended reduced access (5 months and above)	35.9^a^	1.8^b^	5^b^
Extreme temperature	No effect	35.9^a^	8.3^b^	57^c^
	Temporary reduced access (3–4 months)	34^a^	90.8^b^	32^a^
	Extended reduced access (5 months and above)	30.1^a^	0.9^b^	11^c^
Flooding	No effect	28^a^	12.8^b^	50^c^
	Temporary reduced access (3–4 months)	34.4^a^	86.2^b^	35^a^
	Extended reduced access (5 months and above)	37.6^a^	0.9^b^	15^c^
Extreme drought	No effect	24.2^a^	7.6^b^	57.6^c^
	Temporary reduced access (3–4 months)	33.8^a^	91.4^b^	31.3^a^
	Extended reduced access (5 months and above)	42^a^	1^b^	11.1^c^

Each superscript letter denotes a subset of community categories whose row proportions do not differ significantly from each other at the 0.05 level.

We further investigated the perceived effect of climate variability and change on availability of essential forest products used in the communities for livelihood. Table 4 shows similarity in proportion of respondents in Makhado (68.2%) and Mutale (56.9%) that believed firewood is becoming scarce as a result of the effect of climate variability and change. The belief that the effect of climate variability and change is reducing the availability of firewood was significant in Makhado (*p* = 0.0000) but not significant in Mutale (0.1096). Similarly, 74% of the respondents in Thulamela significantly believed that climate variability and change were not having negative effect on the availability of firewood (*p* = 0.0000). The difference in the perceived effect of climate variability and change on firewood across the municipalities is similar to the trends observed in the perceived effect of climate variability and change on access to forest. Generally, it appears that forests in Thulamela are more resilient to current climate risks and are in a better condition to sustainably provide rural dependents with essential goods.

**TABLE 4:** Perceived vulnerability of forest products to climate change and variability effect.

Forest product	Effect on product availability	Proportion of respondents (%) in		

		Makhado (*n* = 156)	Mutale (*n* = 110)	Thulamela (*n* = 100)
Firewood	No	31.8^a^	43.1^a^	74.0^b^
	Yes	68.2^a^	56.9^a^	26.0^b^
	Binomial test	0.00*	0.11^ns^	0.00*
Forest fruits and food	No	12.7^a^	40.4^b^	64.0^c^
	Yes	87.3^a^	59.6^b^	36.0^c^
	Binomial test	0.00*	0.04*	0.00*
Timber/construction wood	No	40.8^a^	85.3^b^	45.5^a^
	Yes	59.2^a^	14.7^b^	54.5^a^
	Binomial test	0.004*	0.00*	0.24^ns^
Charcoal	No	59.9^a^	87.2^b^	10.0^c^
	Yes	40.1^a^	12.8^b^	90.0^c^
	Binomial test	0.03*	0.00*	0.00*
Thatch grass	No	24.2^a^	46.8^b^	76.0^c^
	Yes	75.8^a^	53.2^b^	24.0^c^
	Binomial test	0.00*	0.25^ns^	0.00*
Wild vegetables	No	13.4^a^	27.3^b^	66.0^c^
	Yes	86.6^a^	72.7^b^	34.0^c^
	Binomial test	0.00*	0.00*	0.00*
Mushroom	No	20.4^a^	85.3^b^	32.0^a^
	Yes	79.6^a^	14.7^b^	68.0^a^
	Binomial test	0.00*	0.00*	0.00*
Honey	No	22.9^a^	75.5^b^	36.0^a^
	Yes	77.1^a^	24.5^b^	64.0^a^
	Binomial test	0.00*	0.00*	0.00*
Medicinal plants	No	37.8^a^	17.4^b^	73.0^c^
	Yes	62.2^a^	82.6^b^	27.0^c^
	Binomial test	0.00*	0.00*	0.00*
Fodder for livestock	No	22.4^a^	30.0^a^	67.0^b^
	Yes	77.6^a^	70.0^a^	33.0^b^
	Binomial test	0.00*	0.00*	0.00*

Each superscript letter denotes a subset of community categories whose row proportions do not differ significantly from each other at the 0.05 level. Binomial test analysis of yes and no responses by respondents *, significant at 0.05; ns = not significant at 0.05.

In general, the availability of other forest products (Fodder for livestock, medicinal plants, honey, mushroom, wild vegetables, thatch grass, timber, and forest fruits and food) surveyed in Makhado were perceived to have been significantly affected by climate variability and change (*p* = 0.0000). The perception was in the range of 59.9% – 87.3%. In Mutale, the availability of firewood and thatch grass was perceived not to have been significantly affected by climate variability and change (*p* > 0.05). However, the availability of other forest products, such as fodder for livestock, medicinal plants, wild vegetables, and forest fruits and foods, were perceived to have been significantly affected by climate variability and change (*p* < 0.05). The perception ranged from 53.2% to 87.2%. However in Thulamela, only honey, mushroom and charcoal were perceived to have been significantly affected by climate variability and change (*p* < 0.05). These results indicate that forest products in Makhado and Mutale might be more exposed to climate risk than forest products in Thulamela. More specifically, forest products in Makhado seem to be the most exposed to climate risk. This notion is strengthened by Pearson Chi-square test which showed that there is a significant difference in observed variation in the perceived effect of climate variability and change on the availability of forest products between the municipalities (*p* < 0.05).

### Sensitivity of essential forest products to key climatic impacting factors

We have so far gained insight on the respondents’ belief on the effect of climate variability and change on access to forest and availability of forest products in the study communities. However, the perceived sensitivity of each essential forest product to key climatic factors in the study communities is not yet known. In this regard, binary logistic regression was used to examine the sensitivity of forest products to each of the key climatic events. This was done to ascertain whether there are any forest products perceived to be more vulnerable to particular climatic event. The following forest products were tested: woody products (firewood, charcoal, timber or construction wood) and non-woody products (mushrooms, medicinal plants, thatch grass and wild fruits or vegetables).

The result of the analysis showed that the people do not perceive any of the essential forest products to be particularly vulnerable (*p* > 0.05) to either of erratic rainfall and extreme temperature events. However, increased incidence of flooding, and extreme drought were perceived to have significant association (*p* < 0.05) with the availability of essential forest products in the study communities (Tables 5 and 6).

**TABLE 5:** Perceived sensitivity of forest products to increase incidence of flooding.

Parameter	*df*	Estimate	Standard error	Wald Chi-square	Pr > Chi-square
Intercept	1	1.0018	0.3595	7.7652	0.01
Timber or construction wood	1	-0.2737	0.1050	6.7990	0.01

*df*, degrees of freedom.

**TABLE 6:** Perceived sensitivity of forest products to increase incidence of extreme drought.

Parameter	*df*	Estimate	Standard error	Wald Chi-square	Pr > Chi-square
Intercept	1	1.0018	0.3595	7.7652	0.0053
Firewood	1	0.3320	0.1138	8.5023	0.0035

*df*, degrees of freedom.

Most of the surveyed forest products, such as firewood, charcoal, mushrooms, medicinal plants, thatch grass and wild fruits or vegetables, were observed to have no significant association with increased incidence of flooding (*p* > 0.05). Timber or construction was the only product observed to have significant association with flooding event (*p* < 0.05). Increased incidence of flooding is shown to reduce the likelihood that the people will report scarcity of timber or construction wood by 27%. This may imply that the people do not perceive flooding to be a risk to the availability of timber. In general, flooding event was not perceived to be a risk to any of the forest products used for livelihood in the study communities.

However, incidence of extreme drought was perceived to be a risk to availability of firewood alone in the study communities (Table 6). Occurrence of extreme drought event increases the likelihood that the people will experience scarcity of firewood by 33%. This may imply that the people do perceive extreme drought to be a risk to firewood availability.

Apart from firewood, other forest products tested, such as timber, charcoal, mushrooms, medicinal plants, thatch grass and wild fruits or vegetables, were not perceived to have significant association with increased incidence of extreme drought.

### Implications of climate change impact perception for disaster management

Across the entire sampled communities, majority of respondents perceived increased occurrence of climatic and extreme weather events. These climatic events were perceived to have varying effect on access to forests and forest products availability. Observed variation in the perceptions of climate change impacts on forest products and sensitivity of forest products to key climatic impacting factors raises the concern about the ability of rural people to accurately perceive climate change impacts on their livelihood. Specifically erratic rainfall and temperature were perceived to pose no risk to forest-based livelihood, yet these climatic events are empirically proven to have significant impact on forest growth and vitality (Gauthier *et al*. 2014; Lindner *et al*. 2008; Mpandeli & Maponya 2013; Naidoo *et al*. 2013). In addition, in instances where some forest products (e.g. firewood) were perceived to be at peril to some key climatic events, the perception showed contrasting result. For instance, it was surprising to note that the people perceived firewood to be at peril to extreme drought event but did not perceive timber to be at peril to the same event. This contrast suggests that perception-based analysis is insufficient to accurately determine climate change impact on forest-based livelihood and vulnerability of forest products to climate change at rural community level. Another plausible explanation for this contrast could be that the respondents were more observant about products they used daily than those they use periodically. It could also be that the respondents overwhelmingly underestimated climate change impacts in their community. This notion has been argued by Claas *et al*. (2011), who postulated that the general public often rate climate change impacts on an abstract, cognitive level which might lead to an underestimation of climate change hazards. Similarly, Etkin and Ho (2007) argued that in the absence of empirical data (as in the case of climate change issue) there are persistent biases in people’s risk perceptions.

The findings that more forest products in Makhado were perceived to be vulnerable to climate variability and change effect than in Mutale and Thulamela provided insight on possible influencing factor of climatic risk perception in the study community. In Thulamela, where there was abundance of forests that are in good condition, there was the least number of forest products that was perceived to be vulnerable to climate change impacts. In Makhado, although there were forests in good condition, the high population and associated socioeconomic pressure made the people to perceive most forest products to be vulnerable to climate risk. Although Mutale was located in the arid zone with more exposure to adverse effect of climatic event, fewer forest products than Makhado were perceived to be vulnerable to climate risk. Sustainable forest management and forest-based adaptation initiatives in the study communities will hence benefit from paying attention to socio-demographic characteristics of host communities.

### Implications of climate change impact perception for forest-based local livelihoods

In the context of perceived sensitivity of forest-based livelihood to climatic event, it is interesting to note that though the majority of the respondents perceived increasing trend in climatic events, they however did not perceive it to be a risk to forests and forest-based livelihood in their community. In Thulamela, more people perceived that the climatic event do not have effect on their access to forests. In Mutale, majority of people perceived the effect of climatic event on forests to be temporal, while in Makhado there was no clear pattern. Generally, the people are able to perceive climate change impact on non-wood forest product. Hence, honey, fodder for livestock, medicinal plants, wild vegetables, and forest fruits and foods were perceived to be most affected by the climate change. However, the people were unable to accurately perceive climate change impact on woody forest products. For example, the people perception of climate change effect on availability of firewood and timber showed no clear pattern across the municipalities. This has important implication for forest and climate change management initiatives in the area as knowledge and perception of the potential hazards of climate change impacts to forests is likely to affect the people’s engagement (Crona *et al*. 2009; Davis 2011; Davies *et al*. 2008; Van Aalst, Cannon & Burton 2008).

The study findings on the effect of climate change impacts on forest products and sensitivity of each essential forest products to specific key climatic events provided vital information that can be scaled-up and used to ensure that sustainable forest use and management with respect to climate change are maintained. Generally, the finding suggests that perceptions of climate variability and extreme weather impacts on forest-based livelihood are more influenced by socioeconomic pressure than actual manifestation of climatic events. This could be mainly as a result of the growing interaction between climate and socioeconomic pressure that impacts forest use and management in the area. Ensuring resilience of forest-based livelihood in the study communities will also require attention to overall communities’ socioeconomic development.

The slight differentiation across municipalities with respect to perceived effect of climate change on the availability of essential forest products used for livelihood implies the need for area specific approach for climate risk and forest management. The survey of 10 essential forest products (Table 4) commonly used for livelihood in the district especially those that were perceived to have been significantly affected by climate variability and change calls for sustainable management and conservation of these forest products through different forest development initiatives and entrepreneurship development.

## Conclusion

This study used perception-based analysis to examine the effect of climate variability and change on forests and sensitivity of forest-based livelihood to specific climatic events at rural community level in South Africa. It also highlighted the weakness of this method in analysing climate change effect. However, the method is insightful in understanding climate change manifestation at rural community level. The assessment suggests that manifestation of climate variability and extreme weather event is perceived to be increasing in the study communities. Analysis of the perception of these events as a threat to forest products and forest-based livelihood showed mixed results. Extreme drought was perceived to be a significant threat to only firewood availability. However, other climatic events such as erratic rainfall, extreme temperature and flooding were not perceived to constitute threat to any of the forest products used for livelihood. It could be argued that the non-perception of climate change impacts on forest products could mean that the studied communities may actually be more vulnerable to climate change than thought.

The findings also suggest that the perceptions of climate change impact are more influenced by socioeconomic pressure than actual manifestation of climatic events. In addition, we observed that the perceived effect of climate variability and change on forest products used for livelihood varied significantly across the municipalities. Understanding these factors is essential when developing initiatives for climate risk management through forest management in the study communities. Forest development initiatives that target identified vulnerable forest products in each area can deliver immediate and effective forest and climate change management benefits to the study communities.
